# A Commentary on Parent–Child Cognitive Learning Interaction Research: What Have We Learned from Two Decades of Research?

**DOI:** 10.3389/fpsyg.2016.01210

**Published:** 2016-08-31

**Authors:** Yvette R. Harris, Seham Almutairi

**Affiliations:** Department of Psychology, Miami University, OxfordOH, USA

**Keywords:** coding strategies, parent–child interaction, memory problem solving book reading

## Abstract

The role of family influences on preschool and school age cognitive development has received considerable empirical attention from cognitive developmental psychology researchers in the last few decades. As a result of the interest, investigators have focused their attention on developing coding/observational systems to capture the interactions occurring between mothers and their young children. This paper reviews a select body of research on parent–child cognitive learning interactions with the goal of determining how the researchers have operationalized the behaviors that occur within learning interactions. The paper concludes with a discussion of the suggestions on next steps for conducting parent–child cognitive learning interaction research in the future.

## Introduction

The role of family influences on preschool and school age cognitive development has occupied the discourse and the research of cognitive developmentalists for more than half a century (Hunt, 1961; Bloom, 1964). One reason for this interest is the realization by scholars of the primacy of the family environment in shaping, facilitating or constraining immediate and long term cognitive and intellectual competencies in young children.

**FIGURE 1:**
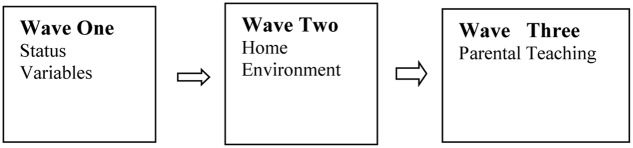
**Conceptual model of family influences on young children’s cognitive and intellectual competencies**.

The ways in which family influences have been operationalized and measured can be conceptualized as representing three empirical waves of research (see **Figure 1**). Each wave posits a different set of questions and at some level is framed from different theoretical and empirical orientations.

**FIGURE 2:**
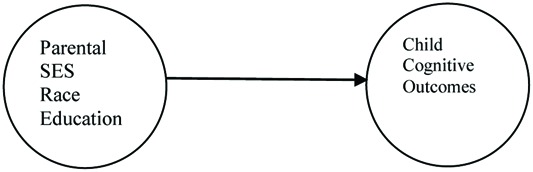
**Conceptual model of early work examining the link between status variables and child cognitive outcomes**.

For example, research conducted during wave one, examines the question of how parental status variables are linked to global measures of preschool and school age intellectual performance. Researchers operationalize status variables as parental educational level, parental socioeconomic status and parental race. This work is motivated by the classic debate surrounding the relative and independent influence of nature versus nurture to children’s intellectual competence (Gottfried, 1984). Numerous studies conducted in the 1960’s and the 1970’s explore the various ways parental socioeconomic status, parental race, and parental educational level predict children’s intellectual functioning (Caldwell and Richmond, 1967; Golden and Birns, 1968; Wachs et al., 1971; Willerman, 1979). **Figure 2** provides an illustration of that relationship.

**FIGURE 3:**
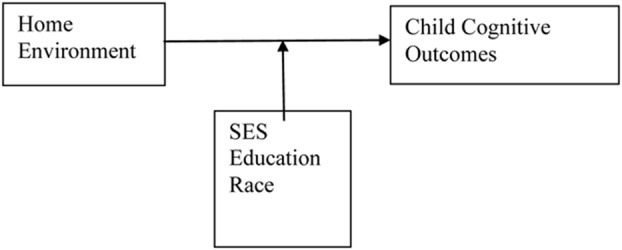
**Conceptual model of research examining the link between home environment and child cognitive outcomes with SES, education race as moderators**.

The second empirical wave concentrates on assessing specific aspects of the home environment (i.e., provision of play materials and parent–child stimulation), and correlating those aspects of the home environment with global measures of children’s cognitive performance (Bradley and Caldwell, 1978). Researchers argue that exploring status variables provide little specificity in illuminating precisely how the home environment provides stimulation to developing children. This work is influenced by the theoretical frame and empirical work of Hunt (1961) and Bloom (1964). Consequently measurements of the home environment were constructed with the goals of identifying and quantifying the overall quality of the home environment for learning (i.e., Bradley and Caldwell, 1981; Caughy et al., 2002). Parental socioeconomic status, education level, and race were put forward as moderators of the relationship between the home environment and child cognitive outcomes. This relationship is shown in **Figure 3.**

**FIGURE 4:**
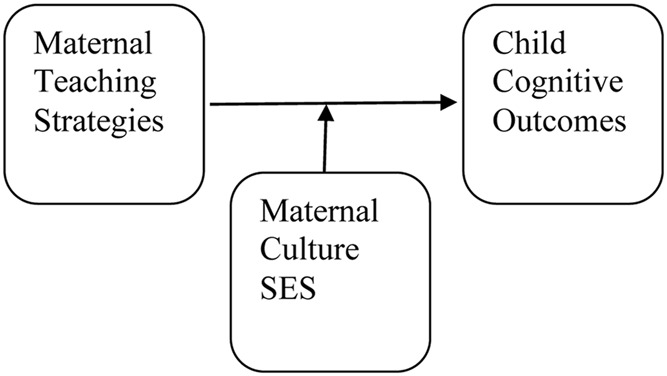
**Conceptual model on research exploring the link between maternal teaching strategies and child cognitive competence with maternal culture and SES as moderators**.

Wave three, developed out of the growing interesting amongst scholars in exploring specific patterns of teaching occurring between parents and children in formal and informal settings. Much of this research is framed from a Vygotsky (1978) theoretical perspective, which acknowledges the important role of social interaction in supporting the development of children’s cognitive skills. Family influences are operationalized as parental teaching behaviors and coding systems are used to capture parental teaching strategies. In this case, investigators are explicitly interested in examining the strategies (e.g., questions, comments), parents use during formal and informal learning activities and exploring how those strategies are linked to children’s concurrent and long-term cognitive competence. This research also focuses on identifying the moderating role of culture, ethnicity and educational level (Rogoff, 1993; Harris et al., 1999). See **Figure 4.**

The three empirical waves summarized above describe the extensive ways family influences are defined, and discuss the different approaches taken with regard to measuring the impact of family influences on child cognitive outcomes and performance. In addition, this body of research, collectively, is influential in shaping a new narrative on the role of family influences on preschool and school age intellectual and cognitive development; and is instrumental in moving interaction researchers beyond a dichotomous view of the contribution of the role of the family on preschool and school age cognitive and intellectual competence.

For this paper, we chose to review a select body of work on parent–child cognitive learning interactions (PCCLI) conducted in the past 20 years (1994–2014) for two reasons. One, during the latter part of the 20th century, the bulk of the work on family influences on children’s cognitive and intellectual performance centers on PCCLI. Two, our research on PCCLI, is informed by the theoretical frame of Vygotsky (1978), and the empirical work of PCCLI researchers.

We had three objectives in mind when assembling this paper. First, we wanted to learn how PCCLI are conceptualized. More specifically, how are PCCLI operationalized, defined, and coded? Second, we wanted to address the broader issue of identifying the critical cognitive skills children learn as a result of interacting with their parents. Third, we wanted to pinpoint the gaps in the literature and offer suggestions for future directions in the field.

## Search Process

In order to find articles for inclusion in this paper, electronic databases such as PsycLIT, ERIC and Mendeley were searched for articles on parent–child cognitive learning interactions. Key words such as parent–child learning, parent–child cognitive development and parent–child interaction were used as the terms to identify articles. The reference lists of the selected articles were then scanned to find additional relevant publications. Twenty four papers published from 1994 to 2014 on parent–child cognitive interactions were finally selected for inclusion in this paper.

The research reviewed here exemplifies some of the major ways in which PCCLI have been studied over the last 20 years. Not every relevant line of research is included in this paper nor are studies of social development, as well as discussions of intervention programs.

## Summary of Major Findings

### Selection Results

Twenty percent (5) of the articles reviewed focus on parental associations with young children’s memory skills; 33% (8) on shared book reading; and 48% (11) on general cognitive skills.

### Measuring Parent–Child Cognitive Learning Interactions

Multiple coding systems have been employed to measure PCCLI. The studies reported in this paper, differ with respect to the number of coding categories (i.e., a low of three to a high of 25 coding categories). Some studies employ frequency counts, while others convert frequency counts to portions, and some researchers use Likert type scales to capture parent and child behavior. Furthermore, there is a great deal of diversity to the extent to which researchers code the children’s performance. That is, some researchers code the children’s concurrent performance (i.e., questions, off task comments) while others code their independent performance using standardized tests, or global scores.

### Memory Skills

The bulk of the research in this area highlights parental influences on children’s memory for past experiences or past events, with specific attention given to the strategies mothers use to elicit those memories from their children. The studies differ significantly in the number of discussions that mothers engage in with their children about past events and differ with reference to the recency of those events. For example, Melzi et al. (2011) had mothers select six events that the children had recently experienced; Cleveland and Reese (2005) had parents’ select four shared and unshared past events to discuss with their children, whereas Tulviste et al. (2015) had mothers’ select two conversations about past events to discuss with their children. Haden et al. (2009), observed mothers engaging in past conversations with their children ranging from two–three events depending on the child’s age and Chang (2003) had mothers select one event that children had recently experienced. The studies also diverge in terms of how elicitation strategies are defined, and how children’s use of strategies in recalling past events are assessed. These studies also differ in exploring the relationship between maternal elicitation strategies and the children’s performance and their inclusion of mothers and children from diverse socioeconomic, and cultural and racial backgrounds.

## Maternal Elicitation Styles

A few of the studies concentrate on maternal elicitation or reminiscing styles, and address how those styles change overtime and pinpoint how they are linked to children’s recall of past events. For example, Haden et al. (2009), identifies two distinct elicitation/reminiscing styles in a sample of Caucasian middle class mothers as they are engaged in memory conversations when their children were 18, 24, and 30 months. Three general categories of maternal memory conversations are scored. *Elaborations* (questions, statements requesting the child to provide new information about the event), *Repetitions* (requests for children to restate information), and *Confirmations* (acknowledging children’s correct responses). From these three categories, two maternal elicitation styles are identified. High eliciting mothers ask more questions, repeat information from their narratives, and affirm the child’s responses. On the other hand, Low eliciting mothers mostly provide new information to their children and this new information is significantly different from the original event. High eliciting mothers overtime, increase their use of questions, continue to provide new information and repeat old information if requested to do so by their children.

The children’s concurrent performance is determined by their use of memory elaborations (provision of new information about the past event), and their use of memory placeholders (repeating mothers comments). The children are also given the Preschool Language Scale-3. Children of high eliciting mothers increase their memory elaborations comments (providing more information) as well as their use of memory placeholders overtime.

Cleveland and Reese (2005) had the central goals of identifying maternal elicitation styles in a sample of New Zealand middle class mothers. Like Haden et al. (2009) they also address how those styles change over time (at 45 and 65 months) and explore how those styles are linked to the children’s performance. They identified four different maternal styles in their research. Mothers are classified as either High Structure/Controlling. These mothers corroborate the children’s recall of the past event, but they may change the topic of discussion. Some mothers are classified as High Structure/Autonomy Supporting. These mothers also validate their children’s recall of the past event, however, they allow their children to assume a larger role in re-telling the story. A few mothers are classified as Low Structure/Controlling. These mothers fail to validate their children’s recall of the event, and they control the discussion of the events, as their children are not given the freedom to tell the story. Lastly there are mothers who are classified as Low Structure/Autonomy support. Like mothers in the previous category, these mothers do not validate their children’s recollection of the past event, yet, they do permit their children to re-tell the past events with little interruption. According to Cleveland and Reese (2005), maternal elicitation style is fairly stable across time, and children of mothers classified as High Structure/Autonomy Support recall significantly more detail about the memory events at both 45 and 65 months than children of mothers from the other styles.

Likewise, Melzi et al. (2011) look at how maternal narrative styles of middle class American mothers and the narrative styles of middle class Peruvian mothers are linked to children’s recall of past experiences at one time point. In this particular study, mothers’ elicitation styles are classified into two categories. There are mothers identified as *Elicitors*, who use comments to primarily elicit information from their children. This style is mainly adopted by the Peruvian mothers. In contrast there are mothers who are identified as *Constructors* and this style is predominantly characteristic of the U.S. mothers. These mothers elicit recall of past events from their children by requesting information, and provide information to assist their children in re-telling the story.

Children’s concurrent contribution to the interaction is assessed by their use of questions, independent contributions, and their requests for assistance from their mothers to aid in their recall of past events. Children of *Elicitors* offer more independent information about the past event than do children of *Constructors.*

## Specific Narrative/Elicitation Strategies

Chang (2003) examines how the narrative strategies employed by middle class Chinese mothers used to discuss past events with their change over the course of a year. She codes both maternal narrative strategy use and child narrative strategy use along two dimensions. *Narrative Structure* which includes references to actions, events, discussions of the location of the event, references to character dialog, and discussions of how the events ended. *Interaction Coding* which assesses: Topic introduction (questions or statements introducing the past event), Questions (soliciting more detail about the event, repeating previously asked questions or soliciting yes/no responses from the child). Memory prompts (statements requiring more information) Clarification (asking for explanation), and Associative talk (comments connecting past events to present events). Chang (2003) found that mothers decrease their use of such as yes/no focused questions overtime and children increase their contribution to the interaction in the form of associate talk overtime.

Tulviste et al. (2015) examine the elicitation strategies that mothers from diverse cultural backgrounds (German, Swedish, Estonian, and Cameroonian-Nso) use as they engage their children in conversations about two past events. Two separate coding systems are used to capture those conversations. One system examines the structure of the conversations, and includes measures of the Total number of independent clauses, (complete sentences and words) Statement elaborations (comments that provide detail about the past event) and Opened ended questions (probing questions to elicit more detail) and Maternal Conversational Dominance (mother controls the discussion of past events). The second classifies the content of the conversations and includes eight categories. (1) Child (number of times the child is referenced as the focus of the past event) (2) Co agency (the number of times both the mother and children are mentioned as the focus of the event), (3) Mother (references to the mothers as the focus of the event), (4) Non-social content (references to the past event with no mention of person), (5) Social Content (referring to other people in the past event), (6) Actions or external behavior references to specific behavior), (7) Mental states (reference to thoughts or feelings during the event), and (8) Physical characteristics (references to clothing, appearance).

Their principal findings reveal that German mothers use more complete sentences and words in structuring the conversation and similar to Swedish mothers use more statement elaborations. Mothers from Estonia ask more open ended questions and mothers from Cameroonian-Nso exert more maternal dominance. In terms of the content of the conversations, Cameroonian-Nso mothers make their children and other people the center of the past event with a specific focus on actions of the actors. Conversely, Estonian mothers, include themselves and their children as central characters in the event, and make references to how they are feeling and thinking about the event. German mothers, on the other hand, emphasize past events that do not include naming specific people, but their stories contain references to such physical states as what people were wearing (clothing) or what they look like (appearances). The children’s narrative strategies also vary by culture. Similar to their mothers, Cameroonian-Nso children include in their stories references to themselves and others as central characters of the event; and German children make references to Non-social content. Unlike their mothers, Co-agency content is higher amongst Swedish children.

### Shared Book Reading

The studies in this domain vary considerably with reference to number of books used during the interaction, mention of socioeconomic status, and inclusion of parents and children from diverse racial and cultural backgrounds. They also differ to the extent to how parent strategies and child strategies are defined and measured and coded.

## Utterances

Robertson and Reese (2015) focus on the types of extra textual utterances used by middle class Caucasian mothers as they are involved in reading two books to their children, a narrative book *Hemi’s Pet* (DeHamel, 1985a,b) and an expository book, *What is a Fish* (Tu akoi, 2007). Extratextual utterances are defined as *Descriptions* (naming or labeling); *Predictions and Inferences* (infer motives or causality), *Relating to Self* (connecting material to life experiences), *Print Talk* (sounding out words), *Evaluations* (likability of the book), *Confirmation* (acknowledging child’s contribution), *Correction/Clarification* (asking for clarity, redirecting the child), *Metacomments* (expression lack of clarity with the text to the child). Three concurrent child strategies were coded- *Child book related initiated* (child interrupts with question), Child book related (child makes a comment about the text) and *Child non-book related* (not related to book). Children were also administered independent language and literacy measures (Test of Early Language Development, Dynamic Indicators of Early Literacy Skills, Letter Naming), as well as concurrent literacy measures (story comprehension and story re-telling tasks).

Their central findings suggest that maternal use of Prediction and Inferences when reading the narrative book correlate with the children’s story comprehension and letter naming performance; whereas, their use of descriptives when reading the expository book correlated with the children’s expressive vocabulary scores.

In a somewhat similar study, Deckner et al. (2006) explore the utterances middle class Caucasian mothers employ while engaged in reading three books with their children—*Clifford: Where is the Big Red Dog* (Bridwell, 1998); *Mice Squeak We Speak* (DePaola, 1997) and *Biscuit’s Picnic* (Capucilli, 1998). In this study utterances are coded as on task and off task. On task utterances are defined as reading or discussing the book, and discussion utterances are further coded into non-metalingual or metalingual utterances. Non-metalingual discussion utterances include requests from the mothers for the child to point to information in the text (referential points) to turn the page (behavioral directives) and to respond to questions. Metalingual discussion utterances are coded as *Prompts* (asking the child to generate a comment), *Responses* (answering the child request for more information), *Recasting* (repeating or expanding upon what children say) *Scripting* (reading the text with pauses), *Vocabulary Introduction* (emphasizing new words), and *References to Print Elements* (comments about the text). Off tasks utterances are considered those comments with no relevance to the reading interaction.

Three levels of the children’s contribution are assessed as well. Level one, their level of interest, availability, affect, and active participation during the book reading interaction on a 5 point Likert type scale (0 = low interests; 5 = high interest). Level two, their independent language performance measured by the Mullen Scales of Early Learning, (administered to the children at 18 months), the Peabody Picture Vocabulary Test-III and the Expressive Vocabulary Test (administered when the children were 30 and 42 months of age). Level three, their emergent literacy skills (Clay’s Concept’s About Print) assessed at 42 months.

Their most important finding shows that mothers employ more metalingual utterances than non-metalingual utterances, regardless of book type, and those utterances are predictive of the children’s affect, and active participation in the reading interaction. Furthermore the utterances correlate with children expressive vocabulary scores at 30 and 42 months.

Correspondingly, Chang and Huang (2015), observe the utterances that mothers and their children of varying socioeconomic levels express while reading the book, *Good Dog Carl* (Day, 1985). Maternal utterances and child utterances are classified into four general coding categories. (1) *Interaction coding* includes requests for provision of information, questions, evaluation of the situation, attention, clarification, turn taking, and feedback. (2*) Immediate Talk* includes information about the location of the story, naming objects in the story, and solicitations for sequential information. (3) *Non-Immediate Talk* captures maternal inferences about the story, predictions about what will happen in the story, emphasis on real life experiences, emphasis on vocabulary, and general knowledge. (4) *Opportunity Education* –consists of maternal comments about issues of morality and right and wrong behavior.

Their major results indicate that mothers from higher socioeconomic levels in contrast to mothers from lower socioeconomic status groups, are more likely to use book reading utterances that make requests for information about the book from their children; and request their children’s sustained attention during the book reading interaction. They also elaborate more in the form of questions about the text of book and unlike mothers from low ses backgrounds offer inferences about the characters in the books, and describe possible outcomes (predictions). In contrast, mothers from low ses backgrounds are more likely than mothers from high ses groups to use the opportunity of the book reading interaction to instruct their children on issues of morality.

Children from higher ses backgrounds are more likely than children from lower ses backgrounds to provide information about the story when requested to do so by their mothers; while children from lower ses backgrounds are more likely to obey commands to return their attention to the text, than are children from higher ses backgrounds.

## Strategies

Moving beyond looking at utterances, Vandermaas-Peeler et al. (2009) examine the types of guidance strategies and the engagement strategies that mothers from middle class and from lower socioeconomic status levels use while reading two books with their children, *Bunny Cakes* (Wells, 2002) and *Bunny Money* (Wells, 2000).

Guidance strategies are coded as *Teaching* (asking questions, sharing information), *Commands* (directing the child’s behavior), *Praise* (acknowledging the child’s performance), *Social connections* (asking the child questions about preference), and *Building bridges* (connecting the story book information to real life experiences). Engagement strategies, are assessed on a three point Likert type scale where 1 equal’s low engagement and 3 equal’s high engagement. Children’s concurrent performance is coded as child initiated (comments child makes to begin the reading activity).

The researchers found middle class mothers use more teaching strategies overall, and more praise in contrast to mothers from the lower ses level. Whereas, mothers from low ses background use more commands. Mothers and children from middle class background are rated as being more engaged in the reading interaction, than are mothers and children from lower class backgrounds.

McDonnell et al. (2003) look at the discourse strategies that middle class Caucasian mothers use when engaged in reading the book *Show and Tell Day* (Rockwell, 1997), with their children over a 3 weeks time frame. They was particularly interested in exploring how their use of discourse strategies change overtime.

Discourse strategies are operationalized as *Conversation Control* (initiations, responses), *Turn Control* (the extent to how long speakers take turns?), *Contextualization Control* (labeling, counting, paraphrasing, explanations, inferences, prediction, and real world connections), and *Lexical Diversity* (number of different words). The children are administered language (Peabody Picture Vocabulary and Expressive Vocabulary Test) and cognitive measures (Bayley Scales of Infant Development).

Their key results suggest that mothers decrease their activity in the book reading interaction overtime (using fewer Discourse Strategies) and the children become more active participants in terms of the increase in their use of Conversational Control strategies during the book reading interaction.

Using a somewhat different approach to capture maternal strategies and child strategies, Kuchirko et al. (2015) coded the types of questions that mothers from different ethnic backgrounds (African American, Dominican, and Mexican) pose to their 3, 4, and 5 years old children as they are engaged in reading *Frog Where are You?* (Mayer, 1969). Questions are coded as *Referential questions* (asks child for a description or label of book text), *Story specific questions* (child is asked to respond to a book specific question), and *Open-ended questions* (asks the child to make inferences and predictions about the story). Their focus in this research is on answering the questions of how maternal questions vary by ethnicity; how maternal questions change overtime and how those questions correlate with children’s narrative contribution. Children’s narrative contributions are assessed using a story grammar framework (references to settings, problems, internal responses, consequences, actions, and resolution), their responses to mothers’ questions and their spontaneous contribution to the story.

Their work yields three major findings. One, mothers independent of cultural background ask more referential questions and open ended questions when engaging in the book reading activity with 3 years old children. Mothers direct more story specific questions to 4 and 5 years old children. Two, Mexican and Dominican mothers ask more referential questions than African American mothers. Whereas, African American mothers ask more story specific questions than Mexican and Dominican mothers. Three, maternal referential, and story specific questions are associated with children’s narrative contributions at age three; maternal use of referential, and opened end questions are associated with children’s narrative contributions at age four and at age five.

## Maternal Styles

A few of the studies in this domain examine stylistic differences in maternal book reading behavior and tie those stylistic differences to the children’s performance. Britto et al.’s (2006) work, for instance, assesses the link between maternal story telling style and children’s vocabulary and school readiness performance in a sample of low income African American mothers engaged in reading the book, *Sounds I Hear* (Gelbart, 1983) with their children. Mothers’ behaviors are scored according to their use of *Timing of Maternal Talk* (how did mothers, introduce and conclude the book reading interaction), *Decontextualized Language* (going beyond the text) *Expressive language use* (questions, points real life experience); *Labeling questions* (requests the child to name an item in the book), and *Positive feedback* (acknowledgment of the child’s performance). Children are administered a series of tests to tap their expressive and receptive vocabulary and their school readiness skills.

Two maternal story telling styles are identified: Story Readers (rarely talked, just read from text) and Story Tellers (make more decontextualized comments, use a number of different words, ask more labeling questions, and give more positive feedback). They found that children of mothers classified as Story tellers have higher expressive language scores in contrast to children of mothers identified as Story Readers.

Similarly, Caspe (2009) focusing exclusively on Latina mothers, also observed mothers reading *Frog Where are You* (Mayer, 1969) and coded mothers strategies into three storytelling styles. Story builders-labelers use strategies to request narrative information from their children; Storytellers, employ strategies that involve telling the story to their children and controlling the direction of the story. Abridged storytellers request concise information from their children. The children are administered concepts about print, letter identification, a narrative task to assess their independent performance. Measures of their concurrent performance include assessments of their referential, evaluative and advanced utterances.

The primary findings show that a Storytellers style significantly predicts children’s concepts about print; and an Abridged story telling style negatively predicts children’s evaluative comments during the interaction.

### General Cognitive Skills Domain

The studies in the third cognitive domain, general cognitive skills is perhaps the most diverse of all of the areas. The cognitive tasks in this domain consist of numeracy (Anderson, 1997; Bjorklund et al., 2004; Susperreguy and Davis-Kean, 2015); problem solving (Kermani and Brenner, 2000; Jimerson and Bond, 2001; Eisenberg, 2002; Hustedt and Raver, 2002; Prudhomme, 2003; Tamis-LeMonda et al., 2004); dinner table conversations (Ely et al., 2001) and shared scientific thinking (Crowley et al., 2001).

The research varies along several different dimensions. First, according to number of interaction sessions. Anderson (1997), Kermani and Brenner (2000), Crowley et al. (2001), Ely et al. (2001), Eisenberg (2002), and Susperreguy and Davis-Kean (2015) observed parent–child interactions in one session. In contrast, Jimerson and Bond (2001), Hustedt and Raver (2002), Prudhomme (2003), Bjorklund et al. (2004), and Tamis-LeMonda et al. (2004) observe interactions over multiple sessions. Second, the studies differ in their operalization of strategies used by parents and children, their focus on culture, and socioeconomic status and whether they include measures of children’s concurrent and independent performance.

## Numeracy

Using a microgenetic approach, Bjorklund et al. (2004) code the *General strategies* (e.g., prompt, prompting after an error, affirmation, disaffirmation, provide answer), and the *Cognitive directives* (e.g., modeling, instruction, and re-representation) that middle class mothers from diverse cultural backgrounds (Bahamas, Columbia, Germany, Great Britain South Africa, and U.S) use as they are engaged in a numeracy activity and game type activity (game board) with their children. Their major aim is to determine how maternal strategy use varies across activity, to determine how maternal strategy use changes overtime, and to identify the association between maternal strategy use and children’s strategy use (e.g., counting, fact retrieval, guessing strategies).

Findings suggest that maternal use of such general strategies as prompting after the child makes an error, and their use of affirmation, correlate with children’s guessing strategies when working on the math activity. In addition, maternal use of disaffirmation, predicts children’s use of addition strategies, and their use of guessing during the game context.

In a somewhat similar study focusing on math, Anderson (1997) explore the type of math talk that occurs in the homes of middle class Caucasian families as they are involved in several different types of tasks (e.g., assembling blocks, and preschool math worksheets). They had a particular interest in assessing the degree to which both parents and children emphasize such strategies as *Counting*, *Naming Shapes*, *Estimating*, *Adding*, *Measuring*, and *Subtracting* across the tasks.

They found that both parents and children employ counting, naming numbers, estimating, and comparing size type strategies (e.g., which one is bigger or smaller) when engaging with the math worksheets; whereas, they use comparing sizes and adding as strategies (e.g., adding sums) when working with blocks.

Like Anderson (1997), Susperreguy and Davis-Kean (2015) examine the type of math talk that occur in the homes of a sample of middle class mothers and their children; however, their focus is on how math talk emerges in every day conversations and activities. The most common type of math talk centers on counting, time, ordinal numbers, and number games. They did not explore links between maternal behavior and child behavior.

## Problem Solving

Jimerson and Bond (2001) observe the strategies that low income mothers use when engaging their children in one free play and two problem tasks (working with Legos to build a structure assembling a puzzle) over two different sessions. The goal of their research is to identify how mothers “take turns” as they engage with their children in these problem solving tasks. Four coding steps are used to capture turn taking strategies. The first step codes *Conversational Turns*, which include verbal and non-verbal behavior acknowledging when one speaker finishes speaking. The second step, *Contingent Interaction* includes five major categories: (1) Uncodeable Turn (comments cannot be coded), (2) Relevant Turn (comments that focus on a mutual understanding of the task) (3) Irrelevant Turn (comments that are off task, and do not relate to the task), (4) Simultaneous Turn (mother and child speaking at the same time) and (5) Not Applicable Turn (turns that do not apply to the above codes). The third step codes *Length of Turn which includes (1)* Alternating Verbal Turns (back forth exchange between mother and child), (2) Long Verbal Turn (comments on the task by the same speaker), and (3) Non-verbal Turn (nods, handing objects to one another). The fourth step *Failure to leave time for a response* codes how long mothers paused to allow the child to respond. The fifth step codes *Interrupting the Child* (mother takes over before the child completes an activity). They found that across problem solving tasks, and sessions, both mothers and children engaged in taking turns equally. In addition, mothers respond to children in relevant ways, they alternate turns with their children and use fewer long verbal turns.

Tamis-LeMonda et al. (2004) explore how low income mothers of color and low income fathers of color engage their children in playing with a set of toys (e.g., teddy bear, large boat with animals) at 24 and 36 months. Six parenting behaviors such as *Sensitivity* (accurately perceives child’s signals during the interaction), *Positive Regard* (parent demonstrates love and respect for the child during the interaction), *Cognitive Stimulation* (part teaches and expands child knowledge during the interaction), *Intrusiveness* (parent is controlling during the interaction), *Detachment* (parent is under involved), and *Negative Regard* (parent rejects the child) are coded on a seven point Likert type scale where 1 equals very low and 7 equals very high. The children are given the Mental Scale of the Bayley’s’ Scale of Infant Development at 24 and 36 months, and the Peabody Picture Vocabulary Test-III at 36 months.

Their major findings indicate that parental sensitivity increases across age, with a slight decrease in positive regard and cognitive stimulation at 36 months. Sensitivity, Positive Regard and Cognitive Stimulation correlate with child performance across time. Intrusiveness positively correlates with children’s MDI scores at 24 months and their PPVT scores at 24 and 36 months.

A few of the studies turn their attention to exploring maternal scaffolding strategies. For instance, Kermani and Brenner (2000) look at scaffolding strategies that mothers from two cultural groups (American Middle Class mothers and Iranian Middle Class mothers) use as they are engaged in two problem solving tasks with their children. One task involves assembling a set of blocks and the second task involves free play.

Scaffolding strategies are coded as *Promotion of Independence* (child is encouraged to perform on their own) *Explanation* (mothers provide clarity to the task) *Inquiry* (mother asks child to perform an activity) *Verbal hints* (mother provides general suggestions), *Verbal Prompts* (mother provides a specific hint), *Instructional* Directives (mother instructs the child to perform a specific behavior), *Modeling* (mother demonstrated for the child), *Correction* (mother redirects child behavior to another solution) and *Physical Control* (mother performs the task on her own). The three child behaviors coded include Asking for help, Refusal of help and Independent Performance.

Their results indicate that independent of task, American mothers use more inquiry and verbal hints; whereas Iranian mothers use more verbal prompts, directives, modeling, correction and physical control. In terms of the children’s behavior, the children, especially the Iranian children ask for more help. There were no reported correlations between the maternal behavior and the children’s behavior.

Similarly, Hustedt and Raver (2002) focus on coding the scaffolding strategies that low income mothers employ on a problem solving task (inserting a straw into a juice box) and a free play task with their children. However, in their study scaffolding strategies are operationalized as *Strategy Support* (verbal hints and direction maintenance), *Manual Help* (mothers offer to help the child) *Modeling* (mother showed child how to insert straw), *Maternal solution* (mother complete the task), *Recruitment* (mothers focusing children’s attention on the task), *Feedback*, (positive or negative comments), and *Off topic Speech* (comments about another subject not related to the task). Preschoolers’ engagement in the activities are coded as: paying attention, solving the task, following directions, seeking help from mother.

The researchers clustered maternal scaffolding strategies into the five distinct categories ranging from Highly Involved mothers (use more verbal strategies overall) to Less involved mothers (who use fewer verbal strategies). Their key findings indicate that children of highly involved mothers pay more attention to the task and their mothers’ directives, work consistently on the task, and avoid engaging in off task behaviors.

Some studies in this domain code maternal use of distancing strategies. The distancing strategies perspective advanced by Sigel (2002) argues that high level strategies, referred to as demands (such as questions, comments that require the child to plan, speculate infer and problem solve) facilitate cognitive growth and development. Using this framework, Prudhomme (2003) examines the link between maternal distancing strategies (e.g., low, medium, and high cognitive demands) and preschoolers’ memory for events in a sample of middle and upper middle class mothers engaging in a free play activity. Mother’s behaviors are coded according to level of distancing demands. *Low level Distancing Strategies* (the parent asks the child to watch, name or label an object), *Medium Level Distancing Strategies* (the parent asks yes/no questions, and makes general comments about the task), *High Level Distancing Strategies* (parents ask the child to complete the task while providing a series of questions, comments and prompts). Two features of the children’s behavior are coded. Their immediate recall and delayed recall (tasks administered during the second visit) as assessed by their performance on the *Novel Enabling Sequences* (imitating making a rattle from two objects) and *Novel Arbitrary Sequences tasks* (imitating pulling a train, dressing and feeding a teddy bear).

Two chief findings emerge from her work. One, the majority of the mothers use low level distancing strategies when engaging their children in a problem solving activity. Two, maternal use of low level distancing strategies and medium level distancing strategies correlate with children’s performance on the enabling sequence.

Using a distance model perspective to coding her data, Eisenberg (2002) investigates how Mexican American mothers from middle class and working class backgrounds engage their children in two problem solving tasks (building a model with blocks, and baking biscuits). The goals of her study are to identify social class and task differences in maternal use of distancing strategies and child use of distancing strategies. Distancing strategies are defined as: *Verbal Supports* (negative feedback, and positive feedback), *Task Structuring* (general directives to perform an act) and *Distancing Behaviors* (statements and questions). Mothers and children’s behaviors are then classified into three mutually exclusive categories. *Low complexity* (labeling, describing, or demonstrating), *Intermediate complexity* (sequencing, classifying, or comparing) and *High complexity* (inferring casual relations, classifying, or comparing).

Her foremost findings reveal that middle class mothers in comparison to working class mothers offer more verbal support to their children in the form of positive feedback and use more intermediate and high complexity type strategies across tasks. In terms of the children’s performance, children from working class backgrounds ask more questions and offer more task structuring comments, then do children from middle class backgrounds.

When looking at task differences, mothers independent of social class provide more positive feedback, ask more questions, and use low complexity type strategies while working on the block task, and use high complexity type strategies while engaged in the baking tasks. Children, ask more questions about baking biscuits, and use both low level and intermediate strategies while engaged in the baking activity with their mothers. She did not examine the link between maternal strategies and preschool strategies.

## Conversations and Communication in Naturalistic Settings

Crowley et al. (2001) explore how middle class parents engage their children in scientific thinking during a museum visit. Three types of parental behavior are coded (describing evidence, giving directions, and explanation) and their results suggest that parents use strategies that describe the evidence more so than they give directions, or provide explanations during a museum visit. The investigators offer no information on the children’s contribution to the discussion during the museum visit.

Ely et al. (2001) examine the types of conversation strategies that middle class Caucasian families use to elicit dinner table conversation from their children. In this case conversation strategies are coded as *Terms*, (list of words they used during the dinner table conversation) and *Pragmatic strategies* which include Control (speaker attempts to control who speaks) Clarification (listener requests explanation), Elicitation (speaker asks child to recount days activities), and Specification (speaker specifies the content of what they would like the other speaker to say). *Metalinguistic codes* are scored as: Emphasis (speaker explains a comment) Comment (makes a comment on words used during the dinner table conversation), Comment about past speech (speaker makes a comment on past speech), Labeling (speaker labels objects, names), Reported speech (speaker requests compliance with good dinner table behavior), and Inanimate speech (speaker makes reference to previous objects), *Literacy codes* which are Literacy (speaker makes reference to engaging in a written activity) and Incomplete (uncodeable).

Several major findings emerge from this work. First, reported speech, comments, and literacy talk are far more common that other forms of speech occurring during the dinner table conversations. Second, mother talk correlates with children’s talk. That is mothers’ use of language focused terms predicts children’s use of language focused terms during dinner table conversations.

## Discussion

The goal of this paper was to present a selected review of research conducted on PCCLI in the last 20 years. We were specifically interested in answering three questions. (1) How do the researchers operationalize and define parent strategies and children strategies? (2) What critical cognitive skills might children “learn” as a result of interacting with their parents? (3) Lastly, what are the gaps in the research and what are next steps?

### Operationalization of Strategies

Overall, the studies, regardless of cognitive domain, identify what might be considered the essential pedagogical strategies that parents use to structure, guide, maintain, and conclude learning interactions with their children. However, they differ to the extent to which they emphasize each of the pedagogical strategies in their research; and how they define those pedagogical strategies.

It appears that collectively for research in the memory skills domain, prominence is placed on how parents’ guide the interaction (i.e., orientation, location statements, and repetition) and maintain the interaction (i.e., confirmation, future talk, and correction) as they elicit narratives of past events from their children. It also seems that the varied ways in which maternal behaviors have been operationalized in this domain cohere into two distinct categories. Those categories are maternal styles, and specific narrative strategies. Thematically it could be argued that mothers are viewed as information processing supports for their children.

In the book reading domain, the shared variance” among the studies is their observation of how parents’ guide the shared book reading interaction (e.g., provision of information, labels, correction, evaluation, and questions), and how they maintain the interaction (e.g., general knowledge, prompts, personal experiences, and building bridges). Similar to the maternal behaviors identified in the memory skills domain there are stylistic differences that predict children’s active engagement and contribution to the shared book reading activity. Thematically the case can be made that mothers serve a coaches for their children as they find ways to involve them in the book reading interactions.

The studies in the third cognitive domain, general cognitive skills are perhaps the most diverse of all of the areas. In contrast to the other areas, these studies stress the way in which parents initially structure the interaction. In this case structure is operationalized as scaffolding (i.e., strategies to promote independence, verbal statements, directions and modeling, contingency analysis, cognitive assistance). The researchers also give attention to how parents’ guide the interaction (i.e., give directions, explanation, labeling, and describe evidence), and how they maintain the interaction (i.e., number games or songs, sensitivity, turn taking, clarification, and general knowledge). Thematically, mothers and fathers in some instances, serve in the role as co- creators as they engage their children in problem solving, and numeracy related activities.

### What Are the Critical Cognitive Skills That Children “Learn” from Interacting with Their Parents?

We can only speculate that children “learn” a vast array of cognitive skills as a result of engaging in interactions with their mothers. On the one hand, they develop important book reading skills, such as learning ways to comment on text, to elaborate information presented in the text by linking characters with real life experiences, and to develop their vocabulary. Or they learn what Tamis -Lemonda and Song (2013) refer to as the *initiation -reply-evaluation- sequence*. In addition, they learn ways of sharing narratives about past events with others, and in doing so learn the social pragmatics of sharing those events (i.e., turn taking). Lastly, they learn how to allocate their attention, ask complex questions and infer causality as they engage with their parents in dinner table conversations, visits to museums, and other problem solving activities. All of these skills are critical and important cognitive skills as they lay the foundation for later learning in formal academic settings.

### What Are the Gaps in the Research and What Are the Next Steps for This Body of Research?

First, a major gap in the research is the lack of consistent inclusivity and diversity in observations of dyads from racially and economically diverse groups. At present, based on the available research, we know little about the pedagogical skills of parents from diverse cultural and economic backgrounds as they engage their children in cognitive tasks involving museum visits, dinner table conversations, and naturalistic math talk. Thus an important next step and future direction in this line of research is to continue to explore patterns of teaching in economically, racially, and culturally diverse dyads in those cognitive areas. Especially with an explicit focus on looking at within cultural group variation. It could be argued that parents and children from the same cultural communities differ significantly in terms of their patterns of cognitive learning interactions.

Second, the assortment and sheer number of coding systems should be re-evaluated. Few of the coding systems are explicitly grounded in theory and many appear too complicated to replicate. Thus an important next step may involve simplification of existing coding/observation systems. This might necessitate communication and collaboration among researchers to achieve consensus on operationalization of behaviors, and the metrics used to code those behaviors, especially within cognitive domains. Doing so might assist with the translational potential of PCCLI research for parents, teachers and social policy makers.

As we move forward in the future, it may fitting for PCCLI researchers to expand the focus of their questions. For example, those contemporary questions could address:

(1) **Intraindividual differences**. That is, when and how do children benefit from PCCLI’s? Furthermore, how in the context of interacting with their parents, do they *discover* new strategies? How stable are those strategies, and when and how do those strategies become part of their learning repertoire? Microgenetic designs (Siegler, 2016) might be effective in addressing those questions. (2) **Digital Technology**. How do parents engage their children in using digital technology? There are too few studies in this area, thus it is certainly ripe for future work. The small body of available literature suggests that parents use a set of different strategies when engaging their children in technology based book reading activities versus traditional book reading (Lauricella et al., 2014) and (3) **STEM related activities.** While the research is growing is this area, the major limitation is there is a lack of attention given to children and parents from economically and racially diverse backgrounds. Thus a focus of future work chould be to address how parents from these diverse groups involve their children in daily STEM related conversations and activities.

## Author Contributions

All authors listed, have made substantial, direct and intellectual contribution to the work, and approved it for publication.

## Conflict of Interest Statement

The authors declare that the research was conducted in the absence of any commercial or financial relationships that could be construed as a potential conflict of interest.
